# Language outcomes following long-term auditory processing training in individuals with neurodevelopmental disorders: a retrospective case series

**DOI:** 10.3389/fpsyg.2026.1894423

**Published:** 2026-09-01

**Authors:** Işık Sibel Küçükünal

**Affiliations:** Department of Language and Speech Therapy, Faculty of Health Sciences, Gazi University, Ankara, Türkiye

**Keywords:** auditory perception training, autism spectrum disorders, cochlear implant, Down syndrome, intellectual disability

## Abstract

**Background:**

In this retrospective case series, the longitudinal trajectories of receptive language, expressive language, and vocabulary acquisition during auditory processing training (APT) were described for seven individuals with different neurodevelopmental characteristics. The study aims to highlight intra-case developmental patterns rather than to assess the intervention’s effectiveness.

**Methods:**

Archived clinical records of seven participants with follow-up periods ranging from 24 to 48 months were reviewed. For all cases, the Preschool Language Scale-4 (PLS-4) scores at the baseline, 6-month, 12-month, 18-month, and 24-month observation windows were retained as unique age ranges corresponding to the highest item group in which all items were correctly completed at each assessment. Where a participant correctly completed all items in one age group but was unable to complete all items in the subsequent age group, their level was classified as the age group in which they had completed all items. Word counts reflect newly recorded words within each interval. The analyses were conducted on a case-by-case and descriptive basis; no inferential statistical tests were used.

**Results:**

By the twenty-fourth month, all seven cases had progressed to a higher PLS-4 item group age range compared with baseline, in terms of both receptive and expressive language. The number of newly recorded words in the shared observation window also increased at each stage. Whilst the median number of words recorded at baseline was 4 (range: 0–34), this figure was determined to be 678 (range: 133–1,150) for the 18–24-month period. Excluding initial words, the median total number of new words recorded between 0 and 24 months was 1,126 (range: 288–2,238). Individual developmental trajectories varied considerably.

**Conclusion:**

The data indicate that, over the follow-up period, there was progress and an increase in vocabulary acquisition in the age groups corresponding to the PLS-4 item groups at the case level. However, due to the small, clinically heterogeneous sample, the absence of a control group, and concurrent developmental and educational support, the observed changes cannot be causally attributed to APT. The findings are considered to be hypothesis-generating clinical observations for future controlled studies.

## 1 Introduction

Monitoring the linguistic development of individuals with special needs is a topic of prime importance that requires attention. Language development is directly related to cognitive development. In this context, learning one’s native language helps ensure the participation of individuals with special needs in social life.

In the context of Türkiye, the Turkish language is agglutinative, where the meaning is altered by adding affixes to words. For example, the word “eye (göz)” can be derived in different ways, such as “eyeglasses (göz-lük)” and “optician (göz-lük-çü)” (Adalı, 2024). Turkish children develop receptive and expressive language skills by the age of 3, which continue to improve as they grow. By age 3, they can correctly express all sounds in the language and understand speech when listening (Yalçınkaya, 2018). However, individuals with special needs such as autism spectrum disorder (ASD), intellectual disability (ID), and Down syndrome (DS), who have a delay in receptive and expressive language, mainly speak noun words and resort to labeling language. These individuals may have difficulty understanding speech while listening.

A study aimed to increase the language and social skills of primary and secondary school children aged 4–5 to 8–10 years with ASD using robot technology in special education. In this approach, thanks to the content and methods used, children’s language motivation levels increased, their interest in education was enhanced, and their social interactions with their peers improved (Herrero-Martín et al., 2024). Similarly, various educational methods can be applied to correct the delay in receptive and expressive language development. In another study, it is stated that the responses of auditory neurons are affected by attention and learning processes. To correct this effect, it was determined that music facilitates the processes in the auditory system, and music education improves attention and learning processes (Torppa and Huotilainen, 2019). Studies indicate that music education helps improve awareness of frequency, intensity, and duration, and that rhythmic movements in music contribute to the development of language processing skills (Thaut and Abiru, 2010; Weinberger, 1999). Dichotic listening (simultaneous transmission of different speeches to both ears), auditory orientation, and speech comprehension skills under background noise are reported to improve listening ability, thus contributing to receptive language (Majak et al., 2023; Weihing et al., 2015). Consequently, language processing in the Wernicke area, (Bailey and Snowling, 2002; Benasich et al., 2002) will slow down or become complex. In training language comprehension while listening, it is necessary first to provide training in the physiology of the auditory pathway to correctly detect speech sounds while listening to the language. These processes are crucial in understanding the language while listening correctly (Fogerty and Humes, 2012; Medwetsky and Musiek, 2011; Richard G., 2007). Additionally, researchers suggest that auditory memory training can be effective in language acquisition for individuals with Down syndrome. The communication challenges in ASD appear to stem from a different source; reportedly, the communication signal processing in ASD is associated with reduced subcortical sensory functioning in the midbrain (Costa et al., 2015; Schelinski et al., 2022).

Auditory Processing Disorder (APD), also known as Central APD (CAPD), refers to difficulties in processing auditory information in the central nervous system (ASHA, 2005). Over the last two decades, the literature has shown significant variation in both the diagnosis of APD and the efficacy of auditory processing training (APT) therapies. One key subject is the continuous dispute over diagnostic validity, test batteries, and the transfer of training effects to everyday language and academic outcomes. Authoritarian professional organizations (e.g., ASHA, AAA) have released position statements and guidelines emphasizing a test-battery approach, multidisciplinary review, and warning about the therapeutic value of many commercially available auditory training programs (AAA, 2010; ASHA, 2005).

The following synthesis integrates recent empirical work with consensus statements to illuminate current understanding and persistent uncertainties surrounding APT and APD.

Recent systematic reviews and critique papers emphasize that APD is a clinically valid construct only to the extent that assessment and interpretation remain cautious about non-auditory cognitive factors (e.g., language, attention, and working memory) that can influence test performance (De Wit et al., 2016; Miller, 2011). However, the evidence base for targeted auditory interventions producing reliable language or reading gains is limited and frequently mixed when rigorous control conditions are used (Fey et al., 2012; Miller, 2011).

Despite these challenges, one line of research emphasizes the variability of APD presentations, with cluster analysis revealing subgroups that differ across attention, language, and literacy domains. Such subgrouping implies a need for personalized assessment and possibly tailored intervention approaches, rather than a one-size-fits-all APT approach (Sharma et al., 2019).

Richard G. J. (2007) stated that in human hearing and language acquisition, auditory information progresses from the peripheral auditory system to central auditory processing, then through phonemic processing, and finally to linguistic processing. In this context, language problems seen in conditions such as ASD, DS, and ID may stem from a common underlying auditory processing weakness, either before or in addition to diagnosis-specific high-level impairments. The literature on the effectiveness of APT in individuals with special needs is limited to pilot studies and case reports. Given the limited evidence in this field and the fact that most studies are of a pilot or descriptive nature, this study has been designed as an exploratory and retrospective case series aimed at generating preliminary clinical observations rather than establishing causal efficacy. APT is recommended as a supportive intervention focusing on auditory processes in cases of language difficulties. In this context, the study aims to provide preliminary and hypothesis-generating longitudinal clinical observations that could serve as a basis for future controlled research.

## 2 Materials and methods

This study was designed as a single-group, retrospective, and descriptive case series. The study aimed to identify case-specific patterns of change in participants’ language skills over time, based on routine practice records. Language-related outcomes were examined through repeated follow-up assessments. The findings were evaluated within a clinical context characterized by the interplay among developmental maturation, educational experiences, family involvement, and concurrent therapies. They were interpreted as longitudinal observations reflecting the participants’ multi-faceted developmental processes.

### 2.1 Participants

This study employed a retrospective case-series design based on archival clinical records. All participants were selected from the caseload of a private special education clinic, where they had been referred for auditory processing and language rehabilitation. Inclusion criteria were: (a) a confirmed diagnosis of autism spectrum disorder, intellectual disability, Down syndrome, or cochlear implant use; (b) documented receptive and/or expressive language delay; and (c) completion of at least 24 months of APT with regular attendance (≥80% of scheduled sessions). Exclusion criteria were: (a) uncorrected hearing loss other than the cochlear implant user, whose device was properly fitted and monitored; (b) additional neurological or genetic conditions; and (c) incomplete clinical records. All eligible cases meeting the inclusion criteria during the study period (2015–2020) were included. No cases were excluded or lost to follow-up, as the analysis was based on complete records of participants who had already completed the minimum intervention period.

Seven individuals with special needs participated in this study. Among the participants with autism spectrum disorder (ASD), two were female, aged 6 and 20. In the intellectual disability (ID) group, one female participant was aged 20, while the male participant was 6. The participants with Down syndrome (DS) were both male, aged 5 and 6. A hard-of-hearing child was a male cochlear implant user (CIU), 5 years old. Cases received regular APT for at least 24 months between 2015 and 2020. Except for CIU’s mother, all mothers reported that their child had no history of hearing disorders. Written informed consent was obtained from the families of all participants. This work was conducted in accordance with the Code of Ethics of the World Medical Association (Declaration of Helsinki).

### 2.2 Auditory processing training (APT): intervention protocol

The APT protocol was a comprehensive, physiology-based intervention targeting both bottom-up and top-down auditory processing (Chermak and Musiek, 1997). All sessions were conducted individually in a quiet therapy room at a private special education clinic. The training was administered individually by an experienced clinician with extensive experience working with children with special needs, specializing in auditory processing and language rehabilitation. Each session lasted 45 min, twice weekly (1.5 h/week), with a total intervention duration of 24–48 months per participant.

#### 2.2.1 Intervention components

The procedure consisted of four hierarchically structured components, applied successively to each participant based on their developmental and language levels. Progression from one component to the next was determined by the clinician’s clinical judgment based on the child’s mastery of the target abilities.

#### 2.2.2 General developmental training

This component established foundational pre-linguistic skills essential for language acquisition. Structured activities targeted comprehension and labeling of basic concepts, including colors, shapes, numbers, body parts, and facial parts, using concrete, multisensory materials such as toys, household items, and picture cards.

#### 2.2.3 Speed, repetition, and auditory memory training

This component enhanced rapid temporal processing, auditory working memory, and rapid retrieval through interactive games. The core activity was a modified “Which One is Missing?” task (Muluk and Yalcinkaya, 2010), beginning with one object and progressively increasing to five as performance improved. Additional tasks included serial recall (arranging objects in named order) and rapid naming. Object positions were changed rapidly between rounds to increase difficulty, and activities were repeated multiple times per session.

#### 2.2.4 Central auditory processing training

To directly target auditory pathway efficiency, a subset of the Central Auditory Processing Test Battery was adapted for therapeutic purposes (Yalçinkaya et al., 2015). This component included dichotic listening exercises (binaural integration and separation), speech-in-noise listening tasks (figure-ground discrimination), and filtered word listening tasks (recognition of degraded speech signals). Exercises were administered using standardized auditory stimuli delivered via high-quality headphones in a quiet room.

#### 2.2.5 Phonological awareness and higher-level language training

This component focused on metalinguistic skills through syllable segmentation and blending exercises (Semel et al., 2003). Language targets were systematically addressed using a clinician-developed protocol based on the *Clinical Evaluation of Language Fundamentals—Fourth Edition (CELF-4)* and the *5–8 Yaş Dilin Klinik Değerlendirmesi (DKD-4)* (Semel et al., 2003; Yalçınkaya and Belgin, 2007).

#### 2.2.6 Home-based generalization (SUEPL)

To promote generalization, families integrated the SUEPL method into daily routines (Yalcinkaya, 2011), drawing pictures of spontaneous events using colored cardboards and thick writing pens, and repeatedly asking related questions until the child produced target words spontaneously, indicating transfer to long-term memory.

#### 2.2.7 Fidelity monitoring and individualization

Treatment fidelity was ensured through several mechanisms: (a) all sessions were conducted by the same experienced clinician, minimizing therapist-related variability; (b) session content was documented in structured therapy logs following each session; (c) home-based SUEPL activities were reviewed and discussed during weekly clinical sessions to ensure correct implementation; and (d) the clinician monitored each child’s progress systematically to adjust the content and pace of training as needed. The core APT protocol was administered similarly to all participants; however, the pace and duration of each component were tailored to each child’s baseline functioning, developmental level, and rate of improvement. The procedure did not include any diagnosis-specific adjustments because the training was aimed at targeting fundamental auditory processing mechanisms shared by all diagnostic categories.

### 2.3 Data collection tools and process

Data was collected from three primary sources:

#### 2.3.1 Clinical language assessment records

To monitor developmental language skills, the Preschool Language Scale-4 (PLS-4) receptive language (RL) and expressive language (EL) items were administered individually at approximately 6-month intervals (Yalçlnkaya et al., 2007). The results in the archive records indicate the age range corresponding to the highest PLS-4 item group in which the participant correctly completed all items. The participant’s level was determined as the relevant age group (e.g., 18–23 months) only if they correctly completed all items in that age group and were unable to complete all items in the next age group. The original age ranges were retained as ordinal categories. No PLS-4 standardized score, percentile rank, or single age-equivalent score was calculated. For participants above the PLS-4 normative age range, these age ranges were used solely as non-normative descriptive indicators of the language skills demonstrated in the item set.

The PLS-4 is normed for younger children in early language development; when individuals exceed the normative age range, practitioners typically consider the test’s ability to yield valid interpretive information and its congruence with other developmental measures. However, in this study, 20-year-old participants (ASD 2 & ID 1) were assessed using the PLS-4. One of the significant limitations for individuals with neurodevelopmental disorders (e.g., ASD, intellectual disability) is that the vast majority of standard language tests are based on normative values, making it difficult to monitor changes in language skills over time. However, even if age-related norms do not apply to the individual, the test can still provide clinically relevant information. Furthermore, given the limited availability of suitable language assessment tools, particularly for adults with intellectual disabilities, this test is utilized in scientific studies as one of the available appropriate assessment tools. Consequently, the test may be used in clinical practice not only for normative assessment but also to describe an individual’s language profile and to monitor changes in that individual’s language performance over time.

A study on ASD and related outcomes discovered a significant relationship between the Preschool Language Scales (PLS-4) Auditory Comprehension and other cognitive measures in preschoolers with autism spectrum disorder, indicating concurrent validity of language measures with broader developmental assessments in this population (Dempsey et al., 2020). Other research on diverse disabilities (e.g., deaf or hard-of-hearing children with additional disabilities) discusses language outcomes using PLS-4 as part of a larger battery, with findings suggesting variability in language performance by disability type and developmental status, reinforcing the need for type- and context-specific interpretation rather than universal interpretive rules for PLS-4 scores in these groups (Cupples et al., 2018).

#### 2.3.2 Therapy progress notes

The practitioners recorded the words used by each participant during successive assessment intervals. The counts were organized into the following categories: nouns, verbs, adjectives, adverbs, pronouns, negations, conjunctions, and plurals. Each observation value reflects only the words newly recorded during the relevant interval; words counted in an earlier phase are not carried over to the counts of subsequent intervals. Consequently, the interval-specific counts were analyzed separately. Cumulative totals were obtained solely by summing the counts from non-overlapping intervals and were explicitly labeled as cumulative totals.

#### 2.3.3 Semi-structured family interview forms

A semi-structured interview form was developed to gather family-reported observations regarding the child’s developmental history, language status, and intervention experiences. The interview guide explored the following domains: early signs of language delay, diagnostic history, family emotional responses, help-seeking behaviors, family interaction patterns, support resources, perceived challenges, beneficial methods, positive experiences, and advice for other families. Interviews were conducted by telephone or in person at a single time point, during the initial contact with families for study enrollment and informed consent. Each interview lasted approximately 20–30 min. To minimize recall bias, family members were encouraged to provide specific, concrete examples of their child’s daily language use, rather than general impressions.

The central auditory processing training component was feasible for three of the seven participants. However, all seven participants contributed complete data to the main language measures (clinical language assessments, progress notes, and family interviews).

### 2.4 Evaluation

The follow-up records were analyzed across five observation areas. These areas were used to organize clinical data at the case level and were not evaluated as independent tests of the APT’s effectiveness:

#### 2.4.1 Continuity of language development

For each case, the number of new words recorded every 6 months and the cumulative number of distinct words recorded up to the 24th month are summarised.

#### 2.4.2 Receptive language development

Longitudinal changes in the target language were described using the age ranges corresponding to the highest PLS-4 item group, for which all items were completed in each assessment.

#### 2.4.3 Expressive language

Longitudinal changes in expressive language were described using the age ranges corresponding to the highest PLS-4 item group, for which all items were completed in each assessment.

#### 2.4.4 Making connections between words

Therapy notes relating to nouns, verbs, adjectives, pronouns, negation expressions, conjunctions and plural forms were analysed descriptively with the aim of contextualising word counts.

#### 2.4.5 Pragmatic language use

Family reports regarding daily communication, social participation, and functional language use were treated as supplementary qualitative observations.

To enhance reliability, data from the three sources (PLS-4 scores, progress notes, and family interviews) were triangulated to identify consistent patterns of language development across sources. Family interview responses were reviewed thematically, with particular attention to recurring patterns reported by families.

Family reports and clinical follow-up notes further suggested increased independence in daily living skills and continued participation in inclusive education settings. For instance, one mother reported, ‘He now goes to the kitchen and says “want water” by himself,’ while another noted, ‘She follows instructions like “put your shoes on the shelf” without any help.’ Functional use of artistic skills in daily life was also reported; families noted that their children independently engaged in activities such as drawing, beading, and making necklaces or bracelets during free time.”

### 2.5 Statistical analyses

To establish a comparable descriptive framework across cases, the main analyses were based on the baseline and the 6th-, 12th-, 18th-, and 24th-month assessments available for all cases. Records beyond the 24th month were presented at the case level as supplementary data for cases with follow-up. As the assessment timepoints and follow-up periods varied across cases, these records were evaluated within each case’s follow-up period rather than summarised collectively.

The age ranges corresponding to the PLS-4 item groups were treated as ordinal categorical data and reported in their original ranges. Individual trajectories of receptive and expressive language were visually examined, and the number of cases progressing to a higher age group, remaining in the same group, or regressing to a lower group from baseline to 24 months was reported.

The number of distinct words recorded during the initial assessment for each case, along with the number of new words recorded for the first time in each of the 0–6, 6–12, 12–18, and 18–24-month intervals, is summarised separately. In addition, the number of new words recorded during the four follow-up periods after the baseline was totaled to calculate the total number of new words for each case over the 0–24-month follow-up period. To illustrate the distribution across cases, the median, along with the lowest and highest observed values, was reported as a supplementary descriptive measure. In line with the study’s sample structure and research design, the statistical analysis was conducted within a case-based descriptive framework. The PLS-4 results were presented whilst preserving the ordinal categorical structure of the item groups organized by age range; changes over time were examined through individual developmental trajectories, age-specific word counts, and patterns of progress at the case level.

## 3 Results

The findings are presented at the case level for the 0–24-month observation window common to all cases. To enable direct examination of the timing and pattern of change, all five common assessment points are included in the figures.

The characteristics of the cases, including diagnosis, chronological age, and available follow-up periods for each outcome variable, are presented in Table 1.

**Table 1:** Case characteristics and current follow-up records.

Case	Diagnosis	Gender	Age (years)	PLS-4 follow-up period (months)	Vocabulary recording follow-up period (months)
1	ASD	Female	6	36	36
2	ASD	Female	20	48	48
3	ID	Female	20	36	36
4	ID	Male	6	24	24
5	DS	Male	5	24	24
6	DS	Male	6	24	24
7	CIU	Male	5	42	48

ASD, autism spectrum disorder; ID, intellectual disability; DS, Down syndrome; CIU, cochlear implant user. The follow-up period reflects the most recent record available for each outcome variable and should not be interpreted as a directly comparable treatment duration.

An examination of Table 1 reveals that the cases fall within the 5–20-year age range and represent four distinct clinical groups. Whilst the PLS-4 and word records for Cases 4, 5, and 6 ended at 24 months, records for Cases 1 and 3 extend to 36 months, and those for Case 2 extend to 48 months. In Case 7, however, PLS-4 records continue through the 42nd month, whilst word records continue through the 48th month. Due to this variation, common descriptions across cases have been based on the 0–24-month period, whilst subsequent records have been analyzed at the case level.

The language age ranges corresponding to PLS-4 performance in receptive and expressive language domains at common follow-up points are presented in Table 2.

**Table 2:** Language age ranges corresponding to PLS-4 performance at common follow-up points.

Case	Language domain	Initial	6 months	12 months	18 months	24 months
1	RL	18–23	24–29	36–41	42–47	48–53
1	EL	12–17	18–23	24–29	36–41	42–47
2	RL	12–17	12–17	12–17	18–23	18–23
2	EL	9–11	12–17	12–17	12–17	18–23
3	RL	18–23	24–29	30–35	36–41	42–47
3	EL	9–11	18–23	24–29	30–35	30–35
4	RL	30–35	36–41	42–47	48–53	48–53
4	EL	18–23	18–23	24–29	36–41	48–53
5	RL	12–17	24–29	30–35	36–41	42–47
5	EL	12–17	18–23	24–29	30–35	36–41
6	RL	24–29	30–35	36–41	48–53	54–59
6	EL	18–23	24–29	30–35	42–47	48–53
7	RL	18–23	24–29	36–41	42–47	54–59
7	EL	18–23	24–29	30–35	36–41	48–53

RL, receptive language; EL, expressive language. Values indicate the age ranges for the last item group answered correctly and are not PLS-4 standard scores or single age equivalents. For participants outside the normative age range, these values serve as descriptive rather than normative indicators.

When comparing the baseline with the 24-month mark, all seven cases had progressed to a higher age group in both receptive and expressive language domains; in no case was a 24-month assessment recorded below the baseline level. However, the timing of this progress varied between cases. Whilst a transition to a higher age group was observed at every assessment in Cases 1, 5, 6, and 7, Case 2 exhibited periods where the child remained at the same level across multiple assessments in both language domains. Furthermore, Case 3’s expressive language level and Case 4’s receptive language level remained within the same age group at both the 18- and 24-month assessments. These findings indicate that, whilst the general direction of change is similar, the timing and pattern of progress vary at the case level.

Table 2 includes assessments at baseline and at 6, 12, 18, and 24 months, to provide a common descriptive framework for all cases. As assessments beyond the 24th month varied by case in terms of follow-up periods and measurement points, these are presented separately in Table 3 to illustrate long-term development on a case-by-case basis.

**Table 3:** PLS-4 language age ranges beyond 24 months.

Case	Language domain	30 months	36 months	42 months	48 months
1	RL	48–53	60–65	—	—
1	EL	48–53	54–59	—	—
2	RL	24–29	24–29	30–35	30–35
2	EL	18–23	18–23	24–29	24–29
3	RL	42–47	48–53	—	—
3	EL	30–35	30–35	—	—
7	RL	66–71	72–77	78–83	—
7	EL	54–59	72–77	78–83	—

RL, receptive language; EL, expressive language. The table includes only cases with a PLS-4 record from the 24th month onwards. The values indicate the age range of the last PLS-4 item group for which the pass criteria were fully met at each assessment.

“—” indicates the end of the observation period for the relevant month.

No shift to a lower age group was observed in the extended follow-up records presented in Table 3; the available records indicated either that the same level was maintained or that progression to a higher group occurred. In Case 7, receptive and expressive language levels reached the 78–83-month age group by the 42nd month, whilst in Case 2, longer periods of stable performance were observed. In Case 3, a transition to a higher receptive language group was observed between 24 and 36 months, whilst the expressive language level remained within the 30–35-month group. As extended records were available for only some cases, these patterns have been interpreted at the case level.

When examining the recorded word counts, the word count at each assessment point reflects the words newly recorded during the preceding six-month period and not included in previous periods’ counts. The figures at the case level and supplementary descriptive summaries are presented in Table 4.

**Table 4:** Number of newly recorded words during the 0–24-month period.

Case	Initial	0–6 months	6–12 months	12–18 months	18–24 months	Total (0–24 months)
1	7	72	126	322	606	1,126
2	0	58	94	143	179	474
3	0	24	42	89	133	288
4	4	77	235	563	898	1773
5	2	14	85	286	678	1,063
6	34	183	319	695	1,041	2,238
7	23	133	348	470	1,150	2,101
Median (range)	4 (0–34)	72 (14–183)	126 (42–348)	322 (89–695)	678 (133–1,150)	1,126 (288–2,238)

The initial value is also shown. Each subsequent column represents the number of words newly recorded during that interval, excluding those recorded in previous stages. The final column is the sum of the four intervals following the initial one.

In all seven cases, the number of newly acquired words increased in the consecutive age ranges of 0–6, 6–12, 12–18, and 18–24 months. The median number of age-specific words was 72 (range: 14–183) in the 0–6-month interval, rising to 678 (range: 133–1,150) in the 18–24-month interval. The total number of new words recorded across the four intervals following the initial phase varied between 288 and 2,238 across the cases; the highest totals were observed in Cases 6 and 7, whilst the lowest totals were observed in Cases 3 and 2. Consequently, whilst a common trend of increase was observed across all cases, the magnitude of the recorded word counts varied significantly.

Table 5 below shows the distribution of newly recorded words during the 0–24-month period according to lexical and grammatical categories.

**Table 5:** Distribution of newly recorded words during the 0–24-month period according to lexical and grammatical categories.

Case	Noun	Verb	Adjective	Adverb	Pronoun	Negation	Conjunction	Plural	Total new
1	688	220	100	15	62	24	4	13	1,126
2	172	144	97	25	36	0	0	0	474
3	180	48	22	2	14	16	0	6	288
4	875	527	145	59	39	26	49	53	1773
5	725	160	125	4	21	28	0	0	1,063
6	1,257	353	230	21	126	158	25	68	2,238
7	987	558	247	54	153	65	13	24	2,101

The values represent the total number of words recorded for the first time in the 0–6, 6–12, 12–18, and 18–24-month intervals following the baseline assessment, broken down by category. Words already present at the baseline assessment have not been included in these totals. The final column shows the total for the eight categories.

In Table 5, the ‘noun’ category accounted for the highest number of new entries in all cases, followed by the ‘verb’ category. The distribution across the other categories varied from case to case; due to the marked differences in total counts, the results have been interpreted not as a normative lexical profile but as supplementary descriptions at the case level.

**Figure 1:**
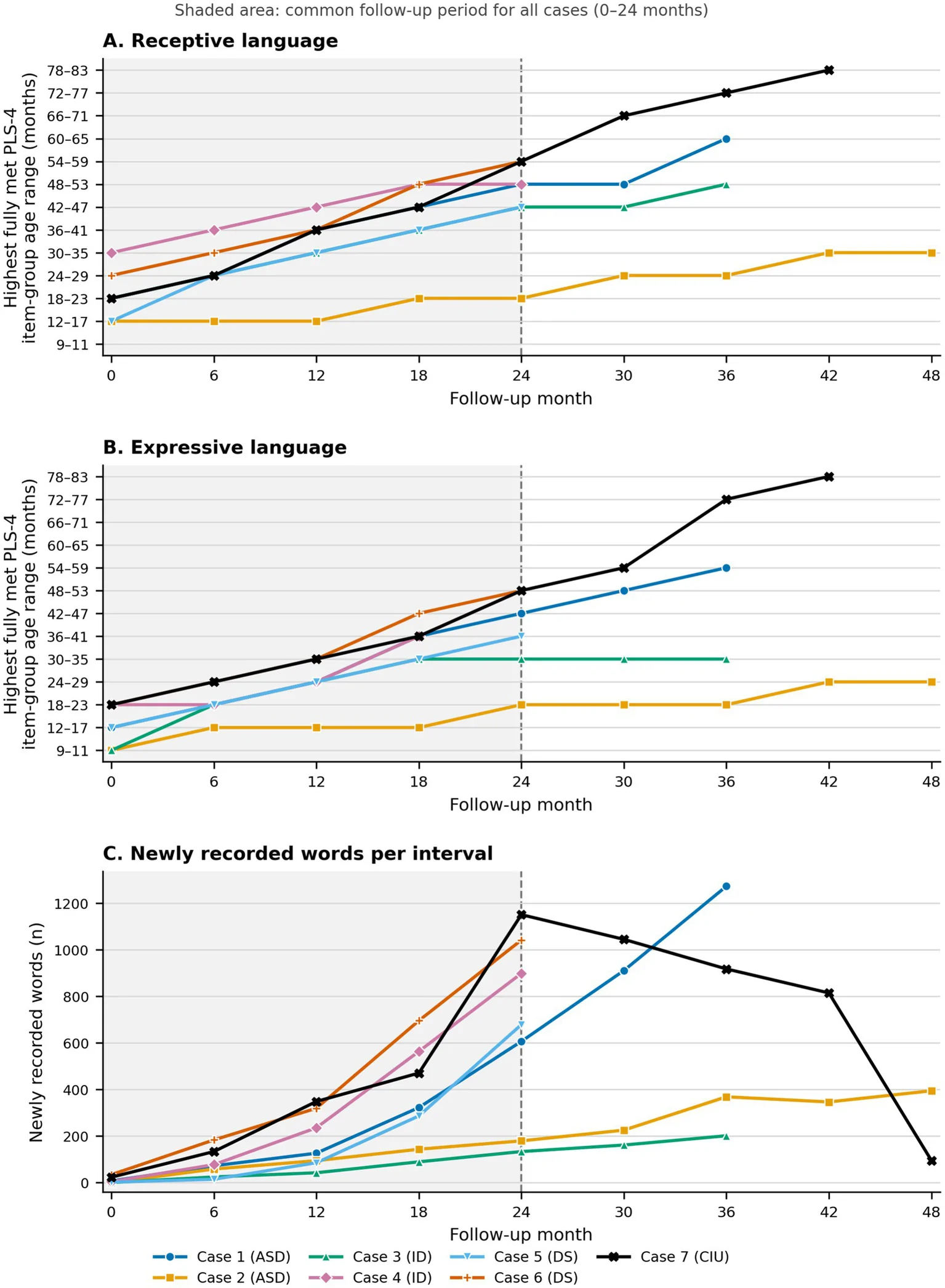
Individual trajectories of receptive/expressive language levels and newly recorded word counts across the follow-up period. The figure consists of three panels **(A, B, and C)** as shown. Panels **A** and **B** show that, during the common 0–24-month follow-up period, cases generally tended to progress to the last PLS-4 item age ranges or to maintain their current level in terms of receptive and expressive language. Panel **C** shows that the number of newly recorded words increased in all cases during the same period, although the magnitude of the increase varied across cases. Follow-up records after the 24th month indicate that progress and stable trends in language levels can be observed alongside increases, decreases, and fluctuations in the number of new words specific to that period. As the follow-up duration and measurement points varied across cases, these long-term trajectories have been interpreted as supplementary descriptive observations at the case level.

Figure 1 below shows individual trajectories of receptive/expressive language levels and newly recorded word counts across the follow-up period.

Panels A and B of Figure 1 show that, during the common 0–24-month follow-up period, cases generally tended to progress to the last PLS-4 item age ranges or to maintain their current level in terms of receptive and expressive language. In Panel C, the number of newly recorded words increased in all cases during the same period, although the magnitude of the increase varied across cases. Follow-up records after the 24th month indicate that progress and stable trends in language levels can be observed alongside increases, decreases, and fluctuations in the number of new words specific to that period. As the follow-up duration and measurement points varied across cases, these long-term trajectories have been interpreted as supplementary descriptive observations at the case level.

To illustrate the individual patterns underlying the group data, case summaries are presented below.

### 3.1 ASD 1: Female, 6 years

#### 3.1.1 Therapy process: 36 months

##### 3.1.1.1 Case history

She was assessed at 2 years of age due to delayed speech development.

##### 3.1.1.2 Initial status

At 5 years of age, limited response to name, reduced eye contact, minimal peer interaction, and delayed adaptive functioning were reported. Toilet training had only recently been completed. APT was initiated at age 6.

##### 3.1.1.3 Follow-up observations

During the follow-up period, longitudinal increases were observed in receptive and expressive language-related measures. By the end of the follow-up period, the difference between language age and chronological age had decreased to approximately 2 years. Family reports and clinical follow-up notes further suggested increased independence in daily living skills and continued participation in inclusive education settings. Functional use of artistic skills in daily life was also reported.

### 3.2 ASD 2: Female, 20 years

#### 3.2.1 Therapy process: 48 months

##### 3.2.1.1 Case history

After being diagnosed with autism at 2.5 years of age, the Family sought second opinions for an extended period before accepting the diagnosis.

##### 3.2.1.2 Initial status

At 20 years of age, the participant initiated APT with limited verbal communication and restricted auditory responsiveness.

##### 3.2.1.3 Follow-up observations

During the follow-up period, gradual increases in speech production and auditory responsiveness were observed, beginning with sound production and progressing toward meaningful word use. Family reports and clinical follow-up notes further suggested improved ability to follow simple verbal instructions, increased participation in daily living activities, and reductions in behavioral difficulties over time.

### 3.3 ID 1: Female, 20 years

#### 3.3.1 Therapy process: 36 months

##### 3.3.1.1 Case history

Living in Vienna, she attended a school for children with special needs; however, expressive language development remained limited, and functional verbal communication was minimal. She primarily relied on sign language and prerecorded cassette phrases for daily communication.

##### 3.3.1.2 Initial status

During a family visit to Türkiye, the Family learned about APT, after which she relocated with her mother and initiated APT at 20 years of age.

##### 3.3.1.3 Follow-up observations

During the follow-up period, longitudinal increases were observed in language comprehension and word production. Family reports and clinical follow-up notes further suggested emerging use of two-word verbal expressions, improved comprehension of five- to six-word verbal instructions, increased orientation in familiar environments, and greater participation in structured daily and social activities. At the time of the final follow-up, she was reported to be working in a municipality-supported setting between 07:30 and 14:30.

### 3.4 ID 2: Male, 6 years

#### 3.4.1 Therapy process: 24 months

##### 3.4.1.1 Case history

Speech production emerged at approximately 4 years of age. By age 6, the participant demonstrated comprehension of common everyday verbal expressions; however, expressive vocabulary was limited to approximately four spoken words.

##### 3.4.1.2 Initial status

APT was initiated at age 6. During the follow-up period, longitudinal increases in expressive verbal communication were observed, including the emergence of 3- to 4-word sentence production.

##### 3.4.1.3 Follow-up observations

Family reports and clinical follow-up notes further suggested progressive development in functional communication and academic participation. The participant later acquired basic reading and writing abilities during primary school and was continuing his education at the high school level at the time of the most recent follow-up. However, his reading comprehension skills were still developing.

### 3.5 DS 1: Male, 5 years

#### 3.5.1 Therapy process: 24 months

##### 3.5.1.1 Case history

The participant did not produce humming sounds or syllabic vocalizations until approximately 3 years of age.

##### 3.5.1.2 Initial status

At 5 years of age, APT was initiated when expressive vocabulary was limited to only a few spoken words.

##### 3.5.1.3 Follow-up observations

During the follow-up period, longitudinal increases were observed in functional communication and adaptive daily living skills. Family reports and clinical follow-up notes further suggested increased independence in self-care activities and participation in inclusive educational settings. At the time of the most recent follow-up, the participant was continuing secondary school education as an inclusion student while maintaining social interaction, peer relationships, and daily routines.

### 3.6 DS 2: Male, 6 years

#### 3.6.1 Therapy process: 24 months

##### 3.6.1.1 Case history

Until age 5, the participant received individual and home-based educational support. The Family later sought additional intervention due to limited vocabulary and a reported plateau in speech development.

##### 3.6.1.2 Initial status

APT was initiated when expressive vocabulary was limited to approximately 15–20 spoken words.

##### 3.6.1.3 Follow-up observations

During the follow-up period, longitudinal increases were observed in expressive communication and auditory responsiveness. Family reports and clinical follow-up notes further suggested improved ability to express needs verbally, follow and retain verbal instructions, and participate more independently in daily living and self-care activities. Literacy-related skills also emerged over time. At the time of the most recent follow-up, the participant was 13 years old, attending secondary school as an inclusion student, participating in sports activities, and independently performing simple household and personal care tasks.

### 3.7 CIU: Male, 5 years

#### 3.7.1 Therapy process: 42 months

##### 3.7.1.1 Case history

The participant was diagnosed with bilateral hearing loss through newborn hearing screening and began using hearing aids at approximately 1 year of age.

##### 3.7.1.2 Initial status

Due to progressive hearing loss, a cochlear implant was applied to the left ear at 5 years of age. APT was initiated during the same period and continued for approximately 3.5 years.

##### 3.7.1.3 Follow-up observations

During the follow-up period, longitudinal increases were observed in expressive communication and social participation. Family reports and clinical follow-up notes further suggested improved self-expression, increased peer adaptation, and greater engagement in social interaction contexts. At the time of the most recent follow-up, the participant was 12 years old, attending a public school, maintaining good academic performance, and aiming to receive a certificate of appreciation. He continued to experience some articulation difficulties and occasional communication challenges in noisy environments.

Across cases, family-reported observations commonly suggested increased engagement in everyday language use and social communication contexts during the follow-up period.

## 4 Discussion

This study aimed to examine individual developmental trajectories in language performance and vocabulary acquisition, rather than to evaluate the effectiveness of the intervention itself. The findings reveal distinct patterns of progress across cases, highlighting the heterogeneity often observed in this population. Despite differing neurodevelopmental profiles, receptive and expressive language scores improved across participants over the follow-up period. Beyond quantitative score changes, longitudinal observations revealed noteworthy improvements in daily communication, adaptive functioning, educational participation, and social engagement in several cases.

Although causal conclusions cannot be established in this retrospective observational design, these descriptive findings suggest that long-term follow-up may be associated with observable language-related progress in individuals with complex communication needs. The literature describes various therapeutic approaches commonly used with this population, including those based on multiple intelligences and behavioral education. These approaches are typically implemented to support cognitive, social, and emotional development. In populations with developmental disorders such as ASD, executive functions and language skills are considered to be closely interrelated. Research shows that the linguistic development of these individuals can be gained through studies in language areas such as vocabulary, syntax, and pragmatic skills. In this context, developing language skills of individuals with autism requires learning the structural aspects of language and acquiring skills for social and contextual use of language. Therefore, supporting executive functions is a fundamental factor in facilitating language development. Strengthening the interaction between these two areas enables individuals with ASD to acquire a more effective language learning (Friedman and Sterling, 2019).

Another study reported that the degree of social impairment among children with ASD was more strongly related to pragmatic language profiles than to structural features of language. This study reveals that social interaction and communication difficulties are directly linked to skills related to the use of language and its appropriateness in social contexts. Although structural language skills, such as vocabulary and syntax, are important, it is emphasized that the pragmatic dimensions of language are the main factors determining autistic children’s social skills and their effectiveness in communication. Pragmatic language skills include individuals’ ability to use language based on its context, communicate appropriately according to social rules, and interact with others. Therefore, the importance of special education methods for strengthening pragmatic language skills to support the social development of children with autism is recommended (Dolata et al., 2022). However, improvements in skills differ depending on children’s level of social communication (Miletic et al., 2024). The longitudinal communication-related changes observed in the present ASD cases may be consistent with previous reports suggesting a relationship between auditory processing, pragmatic language use, and social communication functioning. Individuals with IDs have difficulty formulating questions independently and selecting the necessary vocabulary. At the same time, they require more effort in communication due to their lack of language skills. The fact that they generally use short and simple language structures in their dialogues shows that their linguistic expression skills are limited. It is reinforced by poor vocabulary, misuse of words, and deficiencies in linguistic skills such as morphological and syntactic analysis, synthesis, and abstraction. These deficiencies lie in individuals’ developmentally limited mental processing capacities. In addition, their inability to use intonation appropriate to the messages in their speech makes it difficult for them to convey emotional content, making their communication more mechanical and devoid of emotion. In this context, implementing special education strategies and language therapies for developing the language and communication skills of individuals with IDs is very important (Haiash et al., 2024). Within the training methods, the cognitive behavioral therapy approach is generally preferred (Dagnan et al., 2023). Similar communication-related patterns were descriptively observed in the present ID cases, particularly in functional verbal expression and participation in daily communication.

DS may impair the cognitive abilities in individuals and cause significant deficiencies in language development. This generally causes a delayed development of speech and language skills, which also occurs at a slower rate. Therefore, it is important to determine the most appropriate educational method and strategy to support the language development of individuals with DS. In this context, support for practical language skills is crucial to the healthy progress of individuals’ communicative and cognitive development (Nursanti and Purbani, 2022). Evidence in the literature shows that visual images significantly accelerate the rapid recall of word sounds (Ogg et al., 2020; Ogg et al., 2019). Similarly, research has shown that rapid frequency discrimination training significantly improves individuals’ ability to recognize phonemes in words while listening (Lee et al., 2021). The functional communication and adaptive participation gains observed in the present DS cases may support the importance of language-focused longitudinal interventions in this population.

Systematic reviews and discussions on APT usually indicate that the body of high-quality evidence demonstrating long-term, clinically relevant improvements in APD-specific outcomes or classroom performance is minimal. Several reviews show minimal or ambiguous benefits on auditory processing measures, with inconsistent transfer to language or academic outcomes, especially when trials lack sufficient controls or blinding (De Wit et al., 2016; Fey et al., 2012; Miller, 2011).

Furthermore, the literature on training paradigms (e.g., computer-based auditory training programs, dichotic listening training, and temporal processing training) reveals methodological limitations, including small sample sizes, heterogeneous participant groups (suspected vs. diagnosed APD), variable outcome measures, and potential placebo effects. Consequently, conclusions about the efficacy of APT must be tempered, and generalization to daily functioning remains uncertain in many studies (De Wit et al., 2016; Fey et al., 2012; Miller, 2011).

Taken together, these observational findings should be interpreted as hypothesis-generating rather than confirmatory. They highlight the need for well-controlled, prospective studies with larger sample sizes to evaluate the efficacy of APT in this clinical population systematically.

## 5 Limitations and recommendations

This study has limitations and should be interpreted as an exploratory, retrospective case series based on longitudinal, real-world clinical observations. The study was conducted without a control group or randomization, which limits causal interpretation of the findings. It is important to emphasize that the Preschool Language Scale, Fourth Edition (PLS-4) was used as a descriptive measure to characterize the participants’ language profile, despite being normed for children aged 6 to 11. This decision was made since there are no proven standardized language exams for persons with intellectual disability, and other methods have a known risk of floor effects. Thus, the scores are interpreted descriptively rather than as age-equivalent norms. Furthermore, the concurrent initiation of cochlear implantation in one participant and the ongoing educational and social support received by all participants represent additional confounding factors that prevent causal attribution of the observed improvements to APT alone*. T*he relatively small sample size reflects the challenges of conducting long-term longitudinal follow-up in individuals with complex neurodevelopmental conditions in real-world clinical settings. Despite the limited number of cases, the study includes extended observational follow-up periods of 24 to 48 months, supplemented by later family-reported outcomes on current educational participation, communication abilities, adaptive functioning, and daily life skills. These longitudinal observations allowed descriptive evaluation of developmental trajectories over time. In exploratory retrospective case series designs, such detailed long-term clinical observations may provide clinically meaningful preliminary insights that can guide future controlled investigations.

## 6 Conclusion

In conclusion, this retrospective case series provides preliminary observational findings suggesting longitudinal language-related changes across heterogeneous neurodevelopmental profiles during long-term follow-up. Although causal efficacy cannot be established, the observable functional gains noted in several cases highlight the value of further prospective observational research in this population.

## Data Availability

The datasets generated and/or analyzed during the current study are not publicly available due to privacy and ethical restrictions, but are available from the corresponding author on reasonable request.
